# Fluorescent CQD-Doped Styrene Acrylic Emulsion Coating Film with Enhanced Optical Properties

**DOI:** 10.3390/ijms23010060

**Published:** 2021-12-22

**Authors:** Xiaohui Wang, Li Xu, Rui Yang, Runzhou Huang, Haiyan Mao

**Affiliations:** 1 College of Materials Science and Engineering, Nanjing Forestry University, Nanjing 210037, China; wxiaohui1996@126.com (X.W.); njxl@njfu.edu.cn (L.X.); yangrui@njfu.edu.cn (R.Y.); 2 Department of Chemical and Biomolecular Engineering, University of California, Berkeley, CA 94720, USA

**Keywords:** styrene acrylic emulsion, CQDs, coating, wood processing residues

## Abstract

Styrene acrylic emulsions (SAEs) have emerged as a promising material for water-based coatings. However, they are still limited by their own defects in practical applications, poor weatherability, and degradation of performance at lower or higher temperatures. Here, we introduce a facile approach to producing fluorescent carbon quantum dots (CQDs) from wood processing residues and fabricating fluorescent CQD/SAE coating films via emulsion-casting. The addition of the fluorescent CQDs enhanced the optical performance of the CQD/SAE coating films. The fluorescent CQDs were prepared via a hydrothermal approach and were obtained after heating at 180 °C for 6 h at a reaction concentration of 50 mg/mL. The synthesized CQDs resulted in a high fluorescence, and the CQDs had an average size of 1.63 nm. Various concentrations of the fluorescent CQDs were doped into the SAE coating film, which improved its optical properties. We also characterized and discussed the products and then explored their optical properties. This study presents the potential of fluorescent CQD/SAE coating films for applications in anti-counterfeiting coatings, fluorescent adhesives, and papermaking.

## 1. Introduction

Styrene acrylic emulsion (SAE), as a non-crosslinking emulsion with considerable value for industrial applications, is obtained by the emulsion copolymerization of styrene and acrylate monomers. SAEs are available for coatings, papermaking, and adhesives, owing this to their outstanding advantages for nonpollution, nontoxicity, adjustable gloss, transparency, heat resistance, water resistance, and so on. [1,2,3]. However, the SAEs also have some fatal problems that cannot be ignored, hence, some modification strategies are implemented to improve their properties [4]. For example, SAEs are improved by semi-batch emulsion polymerization to be successfully used in water-based coatings, which possesses an excellent stability, viscosity, gloss, and hardness performance [5]. Furthermore, a styrene acrylic emulsion matrix is used for the preparation of sub-ambient radiative cooling coating materials. It provides a novel avenue for constructing coatings with cooling effects, and substantially expands the potential applications in the construction and energy fields [6]. Nevertheless, only a few nanomaterials are used to modify styrene acrylic emulsions. A polymer with a high electromagnetic interference shielding efficiency has been fabricated from the multiwalled carbon nanotubes incorporated into a styrene acrylic emulsion, showing the obtained product as a potential electromagnetic interference shielding coating material on the building surface [7]. Therefore, fabricating the nanomaterial-based styrene acrylic emulsion materials could bring about more exciting properties, as well as promising applications.

Carbon quantum dots (CQDs), a zero-dimensional and quasi-spherical carbon-based nanomaterial, are fascinating fluorescent nanomaterials that are below 10 nm in diameter. CQDs have inspired extensive investigations into their upconversion fluorescence, tunable fluorescence, electrochemical luminescence, absorbance, redox properties, low cost, nontoxicity, and biocompatibility [8,9]. Their excellent intrinsic characteristics have endowed them with applications in a variety of emerging industries, such as energy storage, nanomedicine, fluorescent probes, and environmental protection [10]. Many preparation methods and applications have been presented in the previous literature [11]. Recently, CQDs with unique optical properties have been reported as a key material in anti-aging studies, which could offer us fresh prospects for overcoming material aging. CQDs have been used in attempts to produce cosmetics with antiaging and antioxidant properties. Tannic acid-derived CQDs with high quantum yield, produced via microwave-assisted pyrolysis, exhibit superior antiaging and antioxidant performance at a low dose and provide great potential applications in nanomedicine and cosmetics [12]. Furthermore, a ZnO/CQD coating was fabricated on brackets, presenting outstanding bactericidal properties under natural light. Owing to the upconversion fluorescence, CQDs could convert visible light into UV light to increase the photocatalytic antibacterial activity [13]. However, current CQDs are still limited by low fluorescence and contamination during their preparation. Green synthesis and ideal fluorescence intensity will be the key to the development of CQDs in the future.

The availability and consumption of biomass resources are important for maintaining the balance of nature with societal needs [14]. The use of biomass residues as recyclable materials, rather than as resources that go to waste, has gradually become a mainstream trend for producing energy and reducing environmental pollution. Wood processing residues cause critical environmental and economic issues that urgently need to be solved [15]. Wood flour, an important filler of wood plastic composites, can be fabricated from the wood processing residues, which involves sustainably processing the residues a second time, thereby presenting the potential recycling value of the wood processing residues [16]. Some other wood processing residues include leaves, branches, bark, sawdust, small chips, and more. The utilization of wood processing residues will set the stage for developing wood-based bioenergy.

In this study, we introduce the fabrication of fluorescent CQD/SAE coating films via a facile emulsion-casting approach. The fluorescent CQDs were prepared from wood processing residues via a hydrothermal method and were obtained after heating at 180 °C for 6 h at a reaction concentration of 50 mg/mL. The synthesized CQDs resulted in a high fluorescence when the excitation wavelength was 365 nm, and the CQDs had an average size of 1.63 nm. Different concentrations of the fluorescent CQDs were added to the SAE coating films to further improve the optical properties of the films. We also analyzed the fluorescent CQDs and CQD/SAE coating films and explored their optical properties. Our work is promising for the development of anti-counterfeiting coatings, fluorescent adhesives, and paper.

## 2. Results and Discussion

### 2.1. Analysis of CQDs Made from Wood Processing Residues

#### 2.1.1. Morphology of Fluorescent CQDs

**Figure 1:**
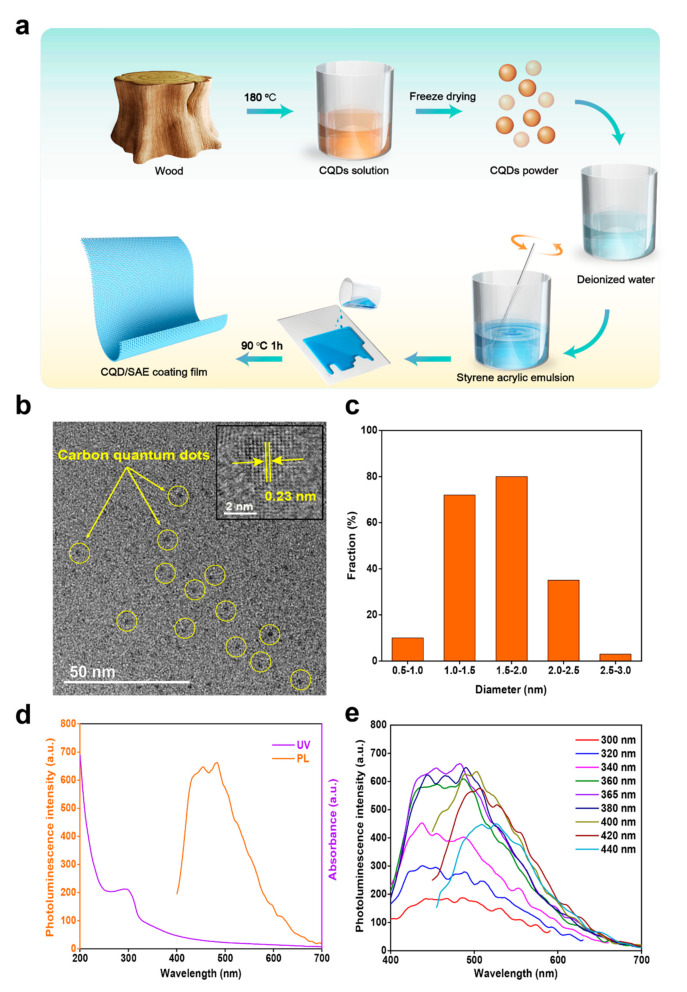
Morphology and optical properties of the fluorescent CQDs. (**a**) The preparation of CQD/SAE coating films. (**b**) TEM image of the CQDs. (**c**) Size distribution of the CQDs. (**d**) UV−vis absorption spectra and PL intensity spectra of the CQDs. (**e**) PL intensity spectra of the CQDs excited at various wavelengths.

We prepared the CQDs from wood processing residues through a hydrothermal method at 180 °C for 6 h. From the TEM image of Figure 1b, we can observe that the spherical CQDs were dispersed uniformly without any agglomeration. In addition, the high-resolution TEM image of the CQDs revealed that the lattice fringes with an interplanar spacing of 0.23 nm were marked by yellow arrows, which indicates that the CQDs possess the crystalline properties of graphitic carbon [17]. We further measured the sizes of the CQDs and obtained an average diameter of 1.63 nm from 200 random particles (Figure 1c).

#### 2.1.2. Optical Properties of Fluorescent CQDs

The optical properties of the CQDs were evaluated depending on their UV-vis absorption and photoluminescence intensity (PL intensity) (Figure 1d). The maximum peak of the UV-vis absorbance was a peak at 294 nm; this peak and its tail were assigned to the n-π* transition of the aromatic sp^2^ C=O bands and the π-π* transition of the conjugated C=C bands from the carbon kernel, respectively. The PL intensity of the CQDs obtained from the wood processing residues by heating at 180 °C for 6 h indicated a fluorescence of up to 663 with an excitation wavelength of 365 nm. Figure 1e presented the typical excitation wavelength-dependent fluorescence properties. The fluorescent emission peaks slightly red-shifted when the excitation wavelength changed from 300 nm to 440 nm, which may be due to the condensation of the CQDs. Moreover, the PL intensity of the CQDs reached its maximum value when the excitation wavelength was 365 nm. This result is consistent with a previous report [18].

We tested the quantum yield (QY) of the wood processing residue-derived CQDs by referring to the method reported in the previous literature [19]. The QY was calculated by an equation wherein quinine sulfate was dissolved in 0.1 mol/L of H_2_SO_4_ ($\varphi_{s}$ = 0.54) as the reference solution.

$$\varphi =\frac{I}{I_{s}}\cdot \frac{A_{s}}{A}\cdot {\left(\frac{\theta }{\theta_{s}}\right)}^{2}\cdot \varphi_{s}$$

where φ is the quantum yield. S is quinine sulfate. I is the integrated area of fluorescence intensity. A is the absorbance, and θ is the refractive index of the solvent (1.3). According to the fluorescence and absorbance of the CQDs and quinine sulfate, the QY of the wood processing residue-derived CQDs is 2.8%. 

### 2.2. Optical Properties of CQD/SAE Coating Films

#### 2.2.1. Photoluminescence Intensity of CQD/SAE Coating Films

**Figure 2:**
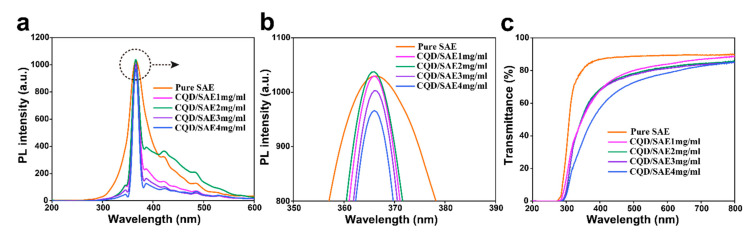
(**a**) The photoluminescence intensity of pure SAE and CQD/SAE coating films. (**b**) The detailed view of PL intensity. (**c**) Transmittance spectra of pure SAE and CQD/SAE coating films.

We prepared transparent CQD/SAE coating films containing different concentrations of CQDs by an emulsion-casting method. The photoluminescence intensity of the prepared coating films was investigated at an excitation wavelength of 365 nm (Figure 2a). We can observe that all the curves show a similar trend, presenting an apparent peak in the range of 360–370 nm, indicating that the addition of CQDs has little effect on the luminescence mechanism of the SAE-based coating film. The PL intensity increased from 350 nm to 365 nm, obviously, and then decreased sharply from 365 nm to 380 nm, which is in the ultraviolet wavelength range (Figure 2b). Finally, the PL intensity tended to be stable in the visible light region. The sole fluorescence emission peak was ascribed to the ester groups and is due to the n-π* electron transition occurring in the oxygen atoms in the free hydroxyl groups on the CQDs and the monomers in the styrene acrylic emulsions. The outstanding fluorescence emission peak was as high as 1030 at an excitation wavelength of 365 nm [20]. Accordingly, we considered that the addition of the CQDs does not affect the location of the fluorescent emission peaks; moreover, a suitable amount of the CQDs could increase the PL intensity of the coating films.

With the addition of CQD concentrations from 0 to 4 mg/mL, the PL intensity gradually increased to the peak and then declined slightly, indicating that the excess CQDs could weaken the fluorescence intensity. As the content of the CQDs increased, the fluorescent CQDs would form aggregations and agglomerations through the van der Waals force, causing fluorescence quenching. Therefore, the excessive CQDs decrease the PL intensity of the CQD/SAE coating films. This result conforms to the findings of a previous study [21]. The reason for this phenomenon is that the doping of the CQDs changes the electron transitions of n-π* and π-π*. The PL intensity reached its peak when the addition of the CQD concentration was 2 mg/mL, suggesting that a proper dose of CQDs could raise the PL intensity of the coating films.

#### 2.2.2. UV Transmittance Spectra of CQD/SAE Coating Films

Figure 2c shows the transmittance spectra of the SAE coating films with various contents of CQDs, which presents a common trend, and indicates that a small amount of added CQDs could still induce visible changes in the styrene acrylic emulsion matrix. The transmittance generally decreased as the content of CQDs increased. When the wavelength range was 300–400 nm, the transmittance increased dramatically until it reached its peak value. The transmittance then gradually tended to be stable at 400–800 nm. The transmittance of the transparent coating films decreased after the addition of CQDs in the visible light region. When the added concentration of the CQDs was 1 mg/mL or less, the transmittance of the coating film decreased by only a small amount compared to that of the pure SAE coating film. We speculate that a small number of CQDs could uniformly distribute in the SAE matrix and had little effect on the transmittance of the SAE coating films [22]. However, when the added concentration of CQDs reached 4 mg/mL, the visible light transmittance of the transparent coating film decreased noticeably, illustrating that the excess CQDs attract each other to form agglomerates and reduce the transparency of the coating films.

Additionally, the maximum value of the CQD/SAE coating films was lower than that of the pure SAE coating film, which implies that the addition of CQDs remarkably enhances the light blocking property of the coating films. Compared to the pure SAE (Ⅰ), the curves of the CQD/SAE coating films (Ⅱ, Ⅲ, Ⅳ and Ⅴ) shifted to the right in the range of 300 nm to 400 nm. The reason for this result was that self-reabsorption occurs as a redshift in the UV−vis absorption spectrum of the added CQDs, which overlaps with their emission peak to ultimately cause further energy loss [23]. Moreover, the rough appearance of the coating films could lead to a light trapping effect and reflect visible light. The process of absorption and re-emission caused a reduction in UV reflection. Therefore, the advantage of CQD addition to the SAE could effectively reduce the UV−vis transmittance of the coating films.

### 2.3. Structural Characterization of CQD/SAE Coating Films

#### 2.3.1. FT-IR Spectra of CQDs and CQD/SAE Coating Films

**Figure 3:**
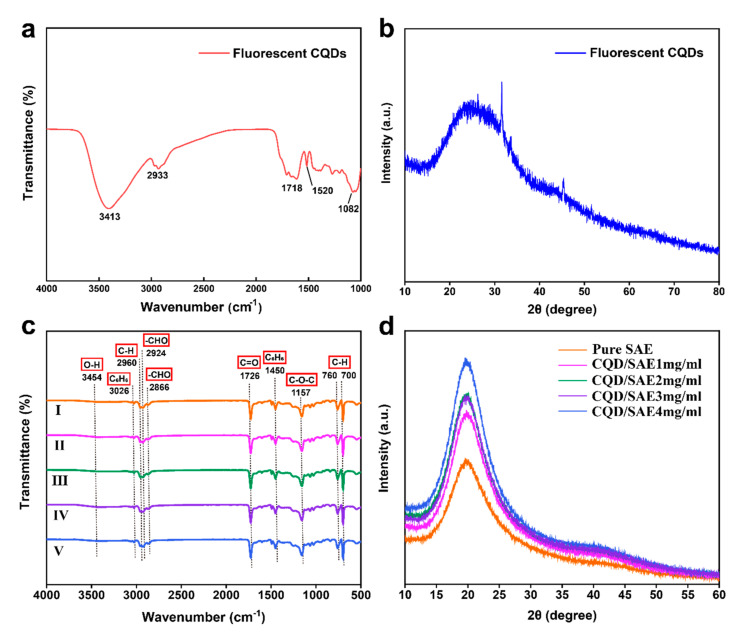
(**a**) FT−IR spectrum of fluorescent CQDs. (**b**) XRD result of fluorescent CQDs. (**c**) FT−IR spectra of pure SAE and CQD/SAE coating films. (**d**) XRD patterns of pure SAE and CQD/SAE coating films.

As shown in Figure 3a, CQDs have a characteristic peak at 3413 cm^−1^, which is attributed to the stretching vibration of the O-H bond. The stretching vibration of the C-H band is represented by the characteristic peak at 2933 cm^−1^. The stretching vibration of carbonyl C=O bonds produces a vibrational band at 1718 cm^−1^ in CQDs. The presence of an aromatic skeleton accounts for the peak at 1520 cm^−1^. The peak of the aromatic C-H bond’s in-plane deformation vibration appears at 1082 cm^−1^. On the surface of CQDs, there are abundant carboxyl groups and hydroxyl groups, which ensures the stability of the CQDs and allows them to be used in subsequent practical applications. 

The FT-IR spectra of SAE coating films with various concentrations of CQDs are presented in Figure 3c. Table 1 lists the locations of the different absorption peaks of the coating films and their corresponding functional groups. The absorption peak at 3454 cm^−1^, that is broad and inconspicuous, was related to the stretching vibration of the O-H bond. The characteristic peaks, uniting at 3026 cm^−1^ and 1450 cm^−1^, were from the styrene monomer of the styrene acrylic emulsion, which was ascribed to the stretching vibration of the benzene ring skeleton. The absorption peak at 2960 cm^−1^ was attributed to the bending vibration of the C-H bands, which is consistent with previous work [24]. The peaks at 2924 cm^−1^ and 2866 cm^−1^ corresponded to aldehyde functional groups. The vibration of the C=O bonds produced a strong stretching vibrational band at 1726 cm^−1^. The stretching vibration of the C-O-C bonds of the esters induced a peak at 1157 cm^−1^, which shows a relatively weak peak owing to electronic transition. The absorption peaks at 760 cm^−1^ and 700 cm^−1^ were associated with the monosubstitution of benzene. The presence of the surface functional groups led to the excellent performance of the CQD/SAE coating films [25].

There was no obvious difference observed among the FT−IR curves of the CQD/SAE coating films, suggesting that the addition of CQDs does not lead to functional group variation in the styrene acrylic emulsion. The basis of the coating films was a styrene acrylic emulsion. In contrast to the spectrum of the pure SAE coating film, no distinct peak appeared at 3454 cm^−1^. However, the broad and significant peak became more evident with the increase in the addition of the CQDs, which illustrates that CQDs were successfully incorporated into the styrene acrylic emulsion. Compared with those of the CQD/SAE coating films, the peaks at 1726 cm^−1^ and 1157 cm^−1^ marginally shifted to the right, which could be due to the presence of a π-π* conjugate effect from the sp^2^ hybrid orbitals of the C-O groups—resulting in the peak value moving in the direction of a large wavenumber [26]. The peaks of the monosubstituted benzene at 760 cm^−1^ and 700 cm^−1^ were caused by the bending vibration of the unsaturated C-H bonds. The out-of-plane deformation vibration of the =C-H bonds also induced these peaks.

#### 2.3.2. XRD Patterns of CQDs and CQD/SAE Coating Films

Figure 3b shows the characteristic peak of the CQD’s diffraction at approximately 23.5°, which may correspond to the diffraction planes of the sp2 graphitic carbon that exactly conforms to the d-spacing of 0.23 nm from the TEM images. Figure 3d presents the XRD patterns of the SAE coating films with various concentrations of CQDs, which exhibit a sharp characteristic peak at approximately 19.7°, suggesting that the CQD/SAE coating film system forms favorably via an emulsion-casting approach. There was also a broad, weak peak at 42.3°. However, the curves had no characteristic CQD peaks, and the crystal structure of the CQDs was not retained in the coating films [27]. The XRD images of the CQD/SAE coating films were similar to each other, which illustrates that the crystal structure of the coating films was almost unchanged, and afterward, the addition of the CQDs only affected the peak intensity of the crystal structure of the styrene acrylic emulsion.

### 2.4. Morphology of CQD/SAE Coating Films

**Figure 4:**
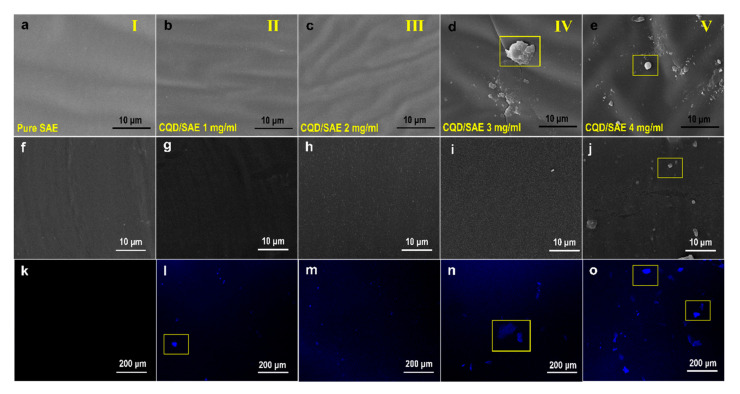
Images of SAE coating films containing various contents of CQDs. (**a**–**e**) SEM images of coating films. (**f**–**j**) Sectional SEM images of coating films. (**k**–**o**) CLSM images of coating films.

The yellowish, transparent SAE coating films containing various concentrations of CQDs were fabricated by emulsion-casting. The surface microstructure of the coating films was evaluated by SEM, as presented in Figure 4a–e, revealing that the coating films had striped surfaces, which were independent of whether the CQDs were added. The appearance of the pure SAE coating film was generally flat. When a small number of CQDs was added to the SAE coating films, the morphology of the coating films exhibited abundant particles that roughened their surfaces, indicating that a small number of the CQDs could disperse uniformly in the SAE coating film matrix [28]. Upon further increasing the addition of CQDs, spherical particles appeared on the surface of the CQD/SAE coating films, which suggests that the excessive CQDs could agglomerate into large particles to form irregular protuberances. We also performed the sectional SEM imaging of the coating films. As shown in Figure 4f–j, the aggregation degree of the CQDs was similar with the SEM images. Moreover, the flatness increased dramatically in the section of the coating films.

The excessive CQDs became tighter with solvent evaporation, indicating that increasing the doping concentration of the CQDs led to poor compatibility between the CQDs and SAE coating film matrix. As various particles were gathered, the monomers of styrene, acrylic acid, and CQDs could attract and wrap their neighboring particles to produce protuberances. However, the surface of the CQD/SAE coating films presented abundant protuberances, which was the result of the effect of various dispersions and aggregation degrees of the CQDs in the SAE matrix. It also illustrated that the incorporation of the CQDs caused a significant change in the surface morphology. This result conforms to the previous work [29].

To further clarify the distribution of CQDs in the SAE coating films, CLSM images were obtained under a 365 nm excitation wavelength, which shows bright blue fluorescence in Figure 4k–o. Fluorescence was not observed from the pure SAE coating film. The blue fluorescence was uniformly distributed with no aggregation when the added concentration of CQDs was 2 mg/mL. However, the CQDs gathered into large particles when the concentration of added CQDs exceeded 2 mg/mL. The appearance of abundant blue fluorescent aggregations was due to the absorbability of the CQDs, which could attract and enwrap neighboring particles. With the increasing CQD content, the blue fluorescence became stronger and more disordered, revealing that the bright PL emission of the CQDs formed from wood processing residues originated from their inherent carbon core of band gaps and extrinsic surface defects [30].

### 2.5. Surface Wettability of CQD/SAE Coating Films

#### 2.5.1. Wetting Property of CQD/SAE Coating Films

**Figure 5:**
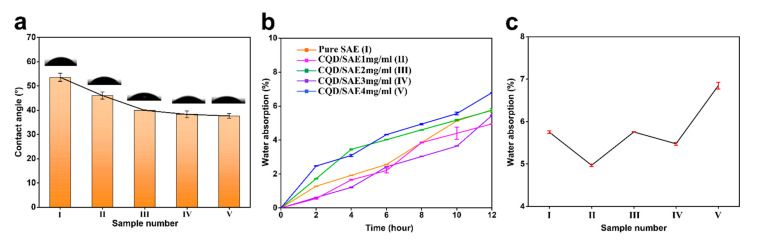
(**a**) Water angle of pure SAE and CQD/SAE coating films. (**b**) Water absorption of pure SAE and CQD/SAE coating films. (**c**) Final water absorption of the various samples.

To explore the surface hydrophobicity of the SAE coating films with different concentrations of CQDs, the water contact angle of the coating films was measured (Figure 5a). The feed rate was 9 μL/drop and 0.5 μL/s. Each sample was measured ten times as a group. The initial contact angle was unstable due to the surface smoothness of the coating film, and the droplet expanded on the film. Therefore, we maintained an equilibrium contact angle. Compared with the pure SAE coating film, the CQD/SAE coating films had lower contact angles, which may be attributed to the introduction of the CQDs. With an increasing CQD amount, the water contact angle decreases. We conjecture that the improvement of the surface wettability is due to the high surface energy, which gathers functional groups on the surface of the coating film—such as carboxyl and hydroxyl groups. Furthermore, the surface roughness of the coating film increased with the addition of the CQDs, and this increase in roughness could be seen from the SEM images. The rough appearance also results in a low water contact angle [31]. This result indicates that the added amount of CQDs has an effect on the surface wettability of the CQD/SAE coating film.

#### 2.5.2. Water Absorption of CQD/SAE Coating Films

To further investigate the wettability of the CQD/SAE coating films, we tested their water absorption. The size of the sample was 2 × 2 cm. The dried coating film was weighed and the mass was recorded as M_0_; the sample was then submerged in deionized water. It was then dried and weighed at room temperature every 2 h, and the mass was recorded as Mn. The water absorption of the coating film was calculated according to the following equation:

Abs = [(Mn − M_0_)/M_0_] × 100%

Water absorption is one of the vital indexes of coating film performance. The lower the water absorption is, the better the water resistance of the coating film. Figure 5b presents the water absorption of different samples after 12 h. Compared with the SAE coating films, the water absorption of the CQD/SAE coating films (1 mg/mL) was lower, which may be ascribed to the introduction of the CQDs. The water absorption then increased slightly with the added amount of CQDs and immersion time. This phenomenon can also be seen in Figure 5c, which shows the final water absorption of the samples after 12 h. We speculate that the surface of the CQDs has carboxyl groups that could absorb many free hydroxyl groups to further decrease the water absorption of the coating film. When the fluorescent CQDs were added to the coating films, the water absorption decreased, indicating that the decrease in hydroxyl groups was caused by a reaction between hydroxyl and carboxyl groups. Furthermore, as more CQDs were added, water absorption was clearly increased. Lower porosity could also lead to lower water absorption [32]. Therefore, it appears to be the result of the decreased water absorption at first. However, with a substantial increase in the CQD amount, the water absorption of the coating film increased remarkably. This result illustrates that the CQD concentration impacts the water absorption of the coating film.

## 3. Materials and Methods

### 3.1. Materials

The CQDs were prepared using wood processing residues as the precursor. The styrene acrylic emulsion (solid content: 40%, viscosity: 80 mPa·s, pH = 8) was purchased from Lin Yi Usolf Chemical Technology Co., Ltd. (Shandong, China). The deionized water was obtained from Su Qian Flexible Materials Technology Co., Ltd. (Suqian, China). All reagents were used as received.

### 3.2. Experimental Methods

#### 3.2.1. Synthesis of Fluorescent CQDs

The CQDs are prepared from wood processing residues via a hydrothermal approach. The dried wood processing residues were ground into powder. The obtained powder (2.5 g) was then mixed with 50 mL of deionized water evenly in a beaker. The solution was then placed in the Teflon-lined autoclave and heated at 180 ℃ for 6 h. The autoclave was cooled to room temperature when the reaction was completed. After that, the obtained product was centrifuged at 10,000 rpm for 20 min to separate the solvent from the residues. The solvent was purified by dialysis for 24 h to acquire the CQD solution. Finally, the CQD solution was frozen overnight and freeze-dried for 48 h to obtain the CQD powder for later use [33].

#### 3.2.2. Preparation of CQD/SAE Coating Films

The CQD/SAE coating films with different contents of CQDs were prepared by emulsion-casting. CQD powders of 0 mg, 5 mg, 10 mg, 15 mg and 20 mg were dissolved in deionized water (5 mL) to obtain the different concentrations of the CQD solutions as follows: 0 mg/mL, 1 mg/mL, 2 mg/mL, 3 mg/mL and 4 mg/mL. The different CQD solutions were poured into a styrene acrylic emulsion (20 mL) with stirring for 1 h. The mixture (SAE emulsion (20 mL) and CQD solution (5 mL)) were then poured on a transparent glass plate and heated at 90 °C for approximately 1 h to obtain the fluorescent coating films with the different concentrations of CQDs (Figure 1a) [34]. The five samples were labeled Ⅰ, Ⅱ, Ⅲ, Ⅳ and Ⅴ, respectively (Table 2).

### 3.3. Characterizations

#### 3.3.1. Characterization of Fluorescent CQDs

To evaluate the morphology of the fluorescent CQDs, transmission electron microscopy (TEM, JEOL JEM-2100, UHR-Ultra High Resolution, Akishima, Japan) was performed to obtain images. The optical performance of the CQDs was investigated using a fluorescence spectrophotometer (LS 55, Perkin Elmer company, Waltham, MA, USA) and a Lambda 950 ultraviolet and visible spectrophotometer (UV-vis).

#### 3.3.2. Characterization of CQD/SAE Coating Films

The optical properties of the CQD/SAE coating films were analyzed using the methods listed above. To explore the structure of the CQD/SAE coating films, Fourier transform infrared spectroscopy (FT-IR) was performed on a German Bruker Vertex 80V spectrophotometer, which scanned from 4000 to 1000 cm^−1^. The X-ray diffraction (XRD) results were obtained by a Rigaku Ultima Ⅳ X-ray diffractometer at a scan rate of 10°/min. Scanning electron microscopy (SEM, Quanta 200, FEI company, Hillsborough, USA) was conducted to study the surface morphology of the CQD/SAE coating films. The distribution of the CQDs in the styrene acrylic emulsion matrix was also tested via confocal laser scanning microscopy (CLSM, LSM710, Oberkochen, Germany). The contact angle was examined by an automatic single fiber contact angle measuring instrument (OCA40, Feldstadt, Germany) to investigate the surface wettability of the coating films. The water absorption was explored by immersing the coating films for various times to estimate their water resistance.

## 4. Conclusions

In this work, a novel CQD/SAE coating film was fabricated with excellent optical properties via an emulsion-casting approach. The fluorescent CQDs were produced using a hydrothermal method and were obtained using wood processing residues by heating at 180 °C for 6 h at a reaction concentration of 50 mg/mL. The different concentrations of the fluorescent CQDs were combined with the SAE coating film, resulting in an improvement in the optical properties of the coating films. The fluorescent CQD/SAE coating film with outstanding optical performance is promising for the development of anti-counterfeiting coatings, fluorescent adhesives, and papermaking.

## Figures and Tables

**Table 1:** Characteristic peaks of the CQD/SAE coating films.

Wavenumber (cm^−1^)	Functional Groups	Vibrations
3454	O-H	stretching
3026, 1450	C_6_H_6_	stretching
2960	C-H	bending
2924, 2866	-CHO	stretching
1726	C=O	stretching
1157	C-O-C	stretching
760, 700	Unsaturated C-H	deformation

**Table 2:** Proportion of experimental materials for the preparation of SAE coating films with various concentrations of CQDs.

Samples	CQD Powders/mg	Deionized Water/mL	SAE/mL
Ⅰ	0	5	20
Ⅱ	5	5	20
Ⅲ	10	5	20
Ⅳ	15	5	20
Ⅴ	20	5	20

## Data Availability

The data presented in this study are available on request from the corresponding author.
